# Sensitivities, Specificities, and Predictive Values of Microbiological Culture Techniques for the Diagnosis of Prosthetic Joint Infection

**DOI:** 10.1155/2014/180416

**Published:** 2014-05-25

**Authors:** Robert W. Jordan, Nicholas A. Smith, Adnan Saithna, Andrew P. Sprowson, Pedro Foguet

**Affiliations:** ^1^University Hospital Coventry & Warwickshire, Clifford Bridge Road, Coventry CV2 2DX, UK; ^2^University of Warwick, Coventry CV4 7AL, UK

## Abstract

*Background*. Identifying the microorganism in a prosthetic joint infection is the key to appropriately targeting antimicrobial treatment. Despite the availability of various techniques, no single test is considered the definitive gold standard. *Aim*. Our aim was to determine the sensitivity, specificity, and positive/negative predictive values for a variety of culture techniques. *Methods*. We performed a retrospective case series of 219 patients undergoing revision surgery of their hip or knee replacement between May 2004 and February 2013. The patients were classified as either infected or noninfected according to criteria set out by the Musculoskeletal Infection Society. The number and type of samples taken intraoperatively varied between cases but included tissue samples and fluid sent in either blood culture vials or sterile containers. *Results*. The highest sensitivity was found with blood culture vials (0.85) compared to fluid in sterile containers (0.26) and tissues samples (0.32). Blood culture vials also had a better specificity and positive and negative predictive values profile. *Conclusion*. We conclude that, of the techniques studied, fluid in blood culture vials had the best profile for the correct identification of microorganisms and advocate its use.

## 1. Introduction

The incidence of infection after primary joint replacement has been reported at 1% to 4% [1–6]. The diagnosis of prosthetic joint infection (PJI) is a challenge as no highly accurate diagnostic method exists. Instead clinicians must rely on a combination of clinical suspicion, serology, culture, and newer molecular techniques [7, 8]. The ability to correctly identify the causative microorganism through culture allows for identification of antibiotic sensitivities and appropriate treatment. Traditional recommendations for microbiological culture suggest taking five or six tissue samples during revision surgery with a definite diagnosis being represented by three or more positive results [9]. However, a number of studies have reported low sensitivities of tissue sampling, [9–11] and a more reliable test for identifying the correct microorganism is desirable.

An improved yield using blood culture vials over tissue sampling and swabs in prosthetic joint infections has been reported [12, 13]. The superiority of this technique in other medical settings such as septic arthritis [14], pleural fluid [15], and spontaneous bacterial peritonitis is reported [16–18]. The aim of our study is to compare the performance of blood culture vials for the intraoperative diagnosis of prosthetic joint infections against the use of sterile containers and tissue sampling.

## 2. Materials and Methods

We retrospectively reviewed 219 consecutive joint revision replacements between May 2004 and February 2013. Patients were classified as either clinically infected (*n* = 33) or noninfected (*n* = 186) according to criteria set out by the workgroup of Musculoskeletal Infection Society (see Table 1) [19]. At our centre the number of neutrophils present on histological specimens is not routinely reported; thus, a positive diagnosis was defined as the presence of one major factor or 3 of the available minor 5 factors.

A single surgeon (PF) performed all the revision procedures in a theatre with laminar airflow. As this was a retrospective study the number and types of samples taken were not standardised and therefore varied between cases. Typically fluid was aspirated and sent for analysis in blood culture vials and a sterile universal container. If only minimal fluid was aspirated then it was placed in one culture medium only. Each blood culture set has one aerobic culture medium and one anaerobic culture medium. The covers are removed and the lids are wiped with an antiseptic swab prior to putting an equal amount of fluid into each bottle (routinely 15 mL each if enough fluid is present). The fluid is then injected directly into each bottle, maintaining an airtight seal. Tissue samples were taken with a fresh sterile scalpel and forceps and put into separate containers. The number of samples taken in each case was again surgeon dependent. Positive growths from enrichment only were not considered positive due to risk of contamination [20]. If the prosthetic joint had been diagnosed as infected, antibiotics were started as soon as the last sample had been taken for the hips and when the tourniquet was let down for the knees.

Electronic microbiological results were retrieved, assessing both the number and types of specimens taken and the number of positive results. Only samples taken intraoperatively were included in the study. In order to help identify any cases incorrectly allocated as noninfected, these patients were followed up after surgery (average 25 months) for any evidence of subsequent infection. This followup entailed review of their clinical records including clinic letters and the need for further treatment. When further surgery was required both the indication and microbiology results were recorded.

Sensitivity, specificity, and positive and negative predictive values were calculated by using standard formulae.

## 3. Results

Of the 219 operations performed during the study period, 146 were revision hip replacements and 73 were revision knee replacements. There were 33 revisions performed for infection, of which 16 were hips and 17 were knees. The average age at the time of surgery was 69.1 years (range 44 to 86 years) and 62.3% were females.  Table 2 illustrates the number of samples taken in each patient group. The sensitivity of fluid in culture bottles was 0.85, compared to 0.26 for fluid in sterile containers and 0.32 in tissue samples. The specificities of all techniques were similar: culture bottles (1.0), sterile containers (0.99), and tissue samples (0.99) (see Table 3). A microorganism was identified in 91% of infected cases with four cases having multiple organisms. The commonest causative organism was coagulase negative* Staphylococcus* as demonstrated in Table 4.

Only 34 patients had results from all three sampling techniques. In this group 15 were total knee replacements and half were performed for infection. 34 results were available for both blood culture vials and sterile containers. A total of 131 tissue samples were obtained in this group, an average of 3.9 samples per patient. The highest sensitivity in this subgroup was obtained when using blood culture vials (see Table 5).

The 186 patients in the noninfected cohort were followed up for a mean of 25 months (range 1 to 93 months). During this period 20 patients (10.8%) required further revision surgery; the indications are shown in Table 6. There were 5 revisions (2.7%) performed for clinically suspected infection and none of these had positive microbiology from their original surgery.

## 4. Discussion

Detecting the correct microorganism is essential for the targeted use of antibiotics following revision in prosthetic joint infections. Traditionally authors have recommended obtaining five or six tissue samples at revision surgery [9]. However, the sensitivity of tissue sampling has been reported between 30% and 61% in the literature [10, 11] and the 32% sensitivity demonstrated in our study raises further concerns over this technique. Various bacteria-related factors such as their paucity in joint fluid, highly fastidious growth, the presence of a biofilm, and the impact of previous antibiotic therapy have been proposed as reasons for these poor results [21]. Therefore, newer techniques have been sought that improve the yield and accuracy of bacterial identification.

Reports have shown that polymerase chain reaction- (PCR-) based methods provide a theoretically more sensitive means of detecting and identifying infectious bacteria [22–24]. Advantages of this technique include faster availability of results, positive results in the presence of only a few copies of bacterial DNA, and the ability to identify nonviable bacteria, for instance, in those patients already on antibiotic treatment. However, some authors have reported high false-positive rates that may occur from contamination either at the time of sample collection or during processing in the laboratory [25, 26]. More recently the use of mass spectrometry in addition to these molecular techniques has been reported to improve yield further [27, 28]. However, due to the limited current knowledge and availability of this technique, conventional cultures remain the most widely used technique in UK practice.

Our data shows an improvement in yield when using blood culture vials compared to tissue cultures in patients with PJIs. This finding is consistent with previous reports in the literature. Font-Vizcarra et al. studied 87 patients with PJI and reported a 90% sensitivity with blood culture vials slightly higher than the 82% sensitivity rate for tissue samples in this group [12]. Similarly Levine and Evans demonstrated in a retrospective review of 24 patients that blood cultures vials had a superior sensitivity (92%) than tissue and swab samples [13]. The 85% sensitivity rate demonstrated in our study further supports the use of this culture modality in PJIs. This ability to improve detection rate has a direct impact on patient care as in these cases antibiotics can be targeted with sensitivities against the known microorganism. This success of blood culture vials is not a novel phenomenon and has been published in other medical settings [15, 16]. Authors have also shown that increasing the number of blood culture vials increases the yield in the diagnosis of bacteraemia [29, 30]. Cockerill III et al. reported 73.2% sensitivity with one set of blood culture vials, 93.9% with two, 96.9% with three, and 99.7% with four [29]. Further studies assessing whether these results can be applied to the field of PJI would be of interest and could improve identification of microorganisms further.

This study has a number of limitations that must be acknowledged. Not all patients undergoing revision surgery had samples taken using all three techniques. Missing data is a common problem in retrospective studies and may introduce bias into the results. Although data for only patients having all three techniques was analysed separately, this only occurred in 16% of patients and these small numbers limit interpretation of these results. The diagnosis of infection was made according to the criteria set out by the workgroup of Musculoskeletal Infection Society [19]; however, at our centre the number of neutrophils present on histological specimens is not routinely reported and the criteria had to be modified accordingly.

Despite the usage of the adapted criteria, it remains possible that a proportion of patients in the noninfected control group had a low grade infection. To investigate this possibility all patients were followed up postoperatively. There were 5 patients in the noninfected control group who required further revision surgery for PJI as per Musculoskeletal Infection Society criteria [19]. This represents a 2.7% risk of infection, which is comparable to infection rates in other revision series [31]. None of these 5 patients had positive microbiology from their original surgery, suggesting that these were likely to be de novo infections.

## 5. Conclusion

The use of blood culture vials was associated with increased sensitivity in identifying microorganisms when compared to fluid in sterile containers or tissue samples. The results of this study suggest that sending fluid in blood culture bottles for microbiological analysis increases the likelihood of correctly identifying the causative organism and we therefore advocate this as standard practice in the investigation of PJI.

## Figures and Tables

**Table 1:** New definition for periprosthetic joint infection: from the workgroup of the Musculoskeletal Infection Society [19].

The presence of a major factor:
(1) Sinus tract communicating with the prosthesis;
(2) Pathogen isolated by culture from 2 or more separate tissue or fluid samples.
The presence of 4 out of the 6 minor factors:
(1) Elevated erythrocyte sedimentation rate and serum C-reactive protein concentration;
(2) Elevated synovial white blood cell count;
(3) Elevated synovial polymorphonuclear percentage;
(4) Presence of purulence in the affected joint;
(5) Isolation of a microorganism in one culture of periprosthetic tissue or fluid;
(6) Greater than 5 neutrophils per high power field.

**Table 2:** Number of patients having at least one sample analysed by each technique.

Sampling technique	Infected cases *N*, (%)	Noninfected cases *N*, (%)
Blood culture vials	20 (60.6)	93 (51.6)
Sterile container	23 (69.7)	99 (53.2)
Tissue sample	31 (93.9)	160 (86.0)

**Table 3:** Sensitivity, specificity, and positive and negative predictive values.

	Sensitivity	Specificity	Positive predictive value	Negative predictive value
Blood culture vials	0.85	1.0	1.0	0.97
Sterile container	0.26	0.99	0.86	0.85
Tissue sample	0.32	0.99	0.88	0.84

**Table 4:** Microorganisms isolated in infected cases.

	Positive standard culture
Coagulase negative *Staphylococcus *	15
*Staphylococcus aureus *	11
*Enterobacter cloacae *	3
*Pseudomonas *	3
*Enterococcus *	2

**Table 5:** Sensitivity, specificity, and positive and negative predictive values for those cases in which all 3 investigations were performed (*n* = 34).

	Sensitivity	Specificity	Positive predictive value	Negative predictive value
Blood culture vials	0.58	1.0	1.0	0.71
Sterile container	0.47	1.0	1.0	0.61
Tissue sample	0.17	0.98	0.93	0.43

**Table 6:** Indication for further revision surgery in the noninfected cohort.

Indication	Number
Instability	11
Infection	5
Periprosthetic fracture	2
Aseptic loosening	2
